# Inositol Metabolism Regulates Capsule Structure and Virulence in the Human Pathogen Cryptococcus neoformans

**DOI:** 10.1128/mBio.02790-21

**Published:** 2021-11-02

**Authors:** Yina Wang, Maggie Wear, Gurkirat Kohli, Raghav Vij, Charles Giamberardino, Arpun Shah, Dena L. Toffaletti, Chen-Hsin A. Yu, John R. Perfect, Arturo Casadevall, Chaoyang Xue

**Affiliations:** a Public Health Research Institute, New Jersey Medical School, Rutgers University, Newark, New Jersey, USA; b Department of Molecular Microbiology and Immunology, Johns Hopkins Bloomberg School of Public Health, Baltimore, Maryland, USA; c Division of Infectious Diseases, Department of Medicine, Duke University Medical Center, Durham, North Carolina, USA; d Institute of Molecular Biology, Academia Sinica, Taipei, Taiwan; e Department of Microbiology, Biochemistry and Molecular Genetics, New Jersey Medical School, Rutgers University, Newark, New Jersey, USA; Yonsei University

**Keywords:** capsule, *Cryptococcus neoformans*, fungal virulence, inositol catabolism, inositol utilization, central nervous system infections, fungal infection

## Abstract

The environmental yeast Cryptococcus neoformans is the most common cause of deadly fungal meningitis in primarily immunocompromised populations. A number of factors contribute to cryptococcal pathogenesis. Among them, inositol utilization has been shown to promote C. neoformans development in nature and invasion of central nervous system during dissemination. The mechanisms of the inositol regulation of fungal virulence remain incompletely understood. In this study, we analyzed inositol-induced capsule growth and the contribution of a unique inositol catabolic pathway in fungal development and virulence. We found that genes involved in the inositol catabolic pathway are highly induced by inositol, and they are also highly expressed in the cerebrospinal fluid of patients with meningoencephalitis. This pathway in C. neoformans contains three genes encoding *myo*-inositol oxygenases that convert *myo*-inositol into d-glucuronic acid, a substrate of the pentose phosphate cycle and a component of the polysaccharide capsule. Our mutagenesis analysis demonstrates that inositol catabolism is required for C. neoformans virulence and deletion mutants of *myo*-inositol oxygenases result in altered capsule growth as well as the polysaccharide structure, including O-acetylation. Our study indicates that the ability to utilize the abundant inositol in the brain may contribute to fungal pathogenesis in this neurotropic fungal pathogen.

## INTRODUCTION

The environmental fungus Cryptococcus neoformans is a major human fungal pathogen that frequently causes life-threatening fungal meningoencephalitis accounting for ∼15% of AIDS-related deaths annually (1, 2). In the absence of treatment, cryptococcal central nervous system (CNS) infections are uniformly fatal (3, 4), and even with aggressive antifungal therapy, the fatality rate can approach 25% (5). Despite its medical significance, our understanding of the molecular basis of cryptococcal neurotropism and the host factors affecting CNS cryptococcosis remain incomplete (6–8). Among the cadre of virulence factors at its disposal, the production of the polysaccharide capsule plays an essential role in the host-Cryptococcus interaction not only by enhancing the yeast’s protection against host stresses but also by modulating host immunity.

The mechanism by which an environmental fungus adapts to the hostile human host environment, resulting in deadly brain infections, is an important issue that remains incompletely understood. Most organisms utilize glucose as their main carbon source, but the bioavailability of this monosaccharide varies and tends to be low in soil and plants where C. neoformans is often found. However, inositol, another monosaccharide, is readily found in the natural reservoir of C. neoformans (9). Our recent work showed that *myo*-inositol (also referred to here as inositol) can stimulate C. neoformans sexual reproduction to complete its life cycle (10). Since both the natural reservoir of this fungus and the human brain are rich in inositol, and inositol clearly affects the C. neoformans life cycle, we were interested to determine if C. neoformans could use inositol as a carbon source and how this would affect virulence.

While most fungal genomes contain one or two inositol transporters, the genomes of C. neoformans H99 and its sibling species Cryptococcus deneoformans JEC21 contain at least 10 inositol transporter (ITR) gene homologs that share functional redundance (11). The expansion of this gene family suggests the importance of inositol utilization in this fungus. Among the products of these genes, Itr1A and Itr3C are low-affinity major inositol transporters required for fungal sexual reproduction and fungal virulence in murine infection models (12). The *itr1a*Δ *itr3c*Δ mutant has a virulence defect with a reduced ability to penetrate across the blood-brain barrier (BBB), suggesting a role for inositol metabolism in fungal transmigration (13). These studies show the genetic capacity for inositol utilization by C. neoformans and indicate that inositol may affect virulence. Taken together, these results led us to hypothesize that C. neoformans has developed an efficient inositol sensing and utilization system and employs this inositol utilization system to promote virulence during CNS infection.

In this study, we examined the role of inositol in capsule growth and the contribution of the inositol catabolic pathway to this process. Our data show that C. neoformans cells grown in inositol-rich medium exhibit enhanced virulence in murine infection models. The genes involved in inositol catabolism were highly upregulated under inositol growth conditions. Importantly, the transcriptome sequencing (RNA-Seq) analyses of yeast isolated directly from human cerebrospinal fluid of patients with cryptococcal meningoencephalitis revealed that inositol metabolic pathways in these yeasts are highly active during CNS infection. Strains lacking inositol oxygenase (MIO) activity, responsible for the conversion of inositol to d-glucuronic acid, show reduced capsule formation in inositol medium and attenuated fungal virulence. Furthermore, comparison of glucose with inositol as the sole carbon source shows that inositol utilization leads to global changes in polysaccharide capsular structure. Our data demonstrate that inositol can be used as a substrate for capsule synthesis in an inositol oxygenase enzyme-dependent manner. Hence, our study reveals a unique pathway for capsule growth, which may contribute to the pathogenesis of CNS cryptococcosis.

## RESULTS

### Inositol-rich conditions enhance virulence of Cryptococcus neoformans.

**FIG 1:**
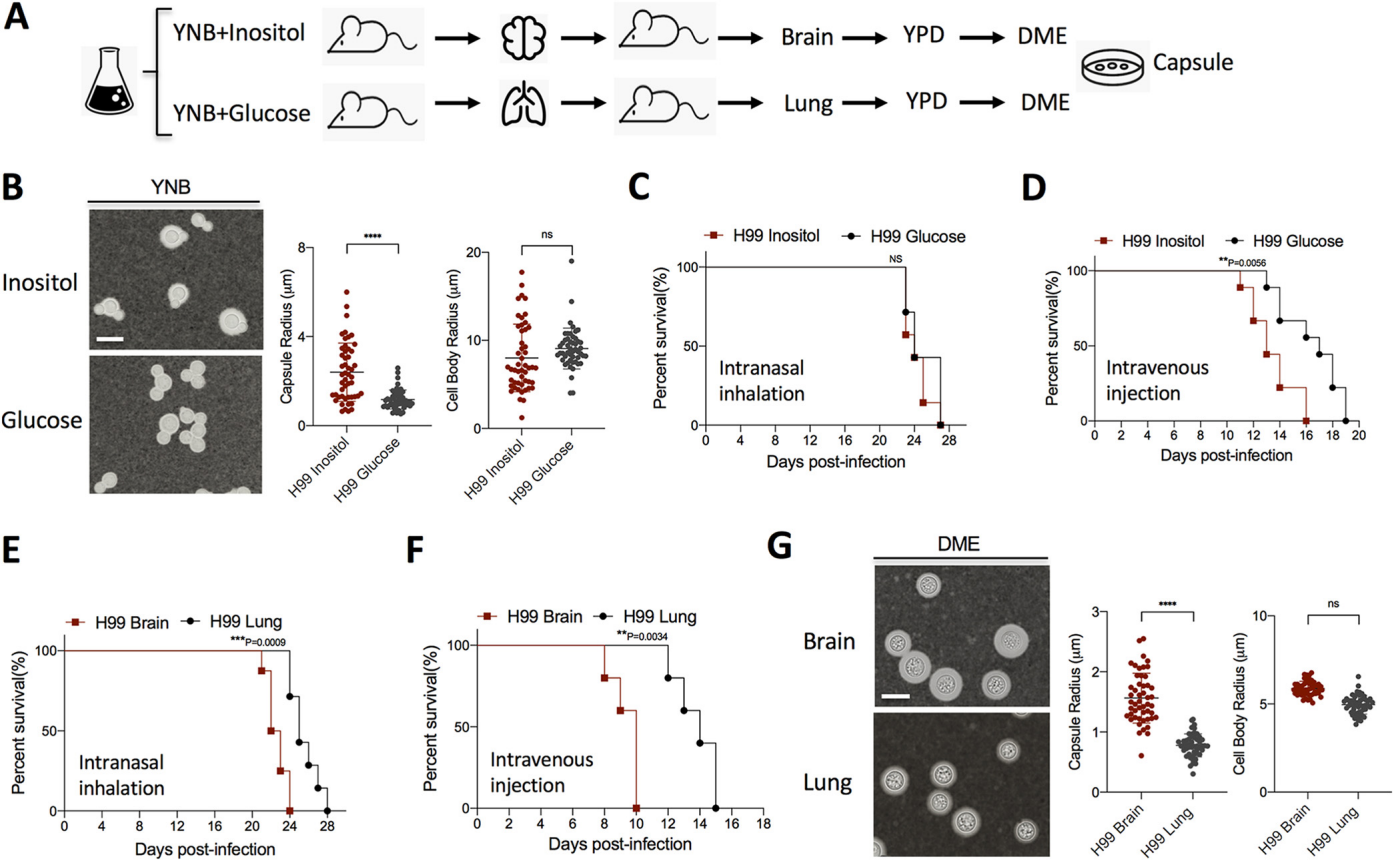
C. neoformans cells grown under inositol-rich conditions exhibit increased virulence. (A) Schematic of experimental design. (B) Cell morphology of H99 after grown on YNB with inositol or glucose as carbon source for 5 days. Quantitative data of both cell size and capsule size from over 50 cells from each sample are presented. (C and D) Survival rates of mice infected with H99 grown on inositol or glucose medium in a murine intranasal inhalation model (C) or intravenous injection model (D). (E and F) Survival rates of mice infected with H99 cells isolated from infected brains or lungs in a murine intranasal inhalation model (E) or intravenous injection model (F). (G) Cell morphology of H99 isolated from infected brain and lung and incubated on DME medium for 3 days. For cell size measurement, representative images are shown. Bar, 10 μm. Statistical analysis was done using a two-tailed *t* test. ****, *P* < 0.0001; ns, not significant. For the animal survival data: ***, *P* < 0.001; **, *P*< 0.01. Statistical analysis was done using a log rank (Mantel-Cox) test.

Previous studies by our group showed that fungal inositol transporters are required for C. neoformans pathogenesis, especially during disseminated infection (10–14). To explore how inositol influences fungal virulence, we examined the morphological and pathogenic differences between glucose- and inositol-grown C. neoformans. C. neoformans is known to utilize inositol as a sole carbon source (15, 16). Yeast cells grown in inositol, while exhibiting slower growth, showed normal development, except for significantly larger capsules. We then sought to determine if the larger capsules observed in inositol conditions affected C. neoformans virulence (Fig. 1A and B). Using the same inoculum size, glucose- and inositol-grown cells were used to infect mice in different murine models of systemic cryptococcosis. While no significant difference was observed in a murine intranasal inhalation model, the animal survival data from a murine intravenous injection model showed that C. neoformans cultured in inositol medium exhibited enhanced virulence compared to cells cultured on glucose (Fig. 1C and D). To determine if the high levels of inositol found in human and animal brains further affect virulence (17), yeast cells isolated from infected brains and lungs were used to reinfect mice. We observed that yeast cells isolated from infected brain caused lethal infection faster than the ones isolated from infected lung of the same animal (Fig. 1E and F). After infection, yeasts isolated from the brain showed significant increases in capsule size compared to yeasts isolated from the lung, suggesting that heritable changes occur following brain passage (Fig. 1G).

To confirm a potential heritable change in inositol-induced capsule change, we also cultured C. neoformans strain H99 on minimal medium with 0.3% inositol or with 0.3% glucose for 10 days or 20 days, and then yeast cells were passaged on yeast extract-peptone-dextrose (YPD) agar medium before inoculated on Dulbecco modified Eagle’s (DME) capsule-inducing medium. Our data showed a significantly larger average capsule size for fungal cells isolated from minimal medium (MM) with 0.3% inositol after more than 10 days incubation (see Fig. S1 in the supplemental material), indicating that the inositol-induced capsule enlargement is heritable, which is consistent with our *in vivo* animal passage results. Taken together, these data support the conclusion that inositol regulates C. neoformans capsule size and influences fungal virulence.

10.1128/mBio.02790-21.1 FIG S1 *In vitro* passage of C. neoformans isolates grown under inositol-rich conditions. (A) Schematic of *in vitro* passage experimental design. (B) Representative images of capsule sizes after India ink staining for the cultures grown on inositol rich minimal medium (MM+Inositol) for different incubation times. (C) Quantification of capsule sizes under culture conditions indicated in panel B. Statistical analysis was done by Student’s *t* test. ****, *P* < 0.0001. Download FIG S1, TIF file, 1.3 MB. Copyright © 2021 Wang et al. 2021 Wang et al. https://creativecommons.org/licenses/by/4.0/ This content is distributed under the terms of the Creative Commons Attribution 4.0 International license.

### Genes involved in inositol catabolism are highly upregulated by inositol.

**FIG 2:**
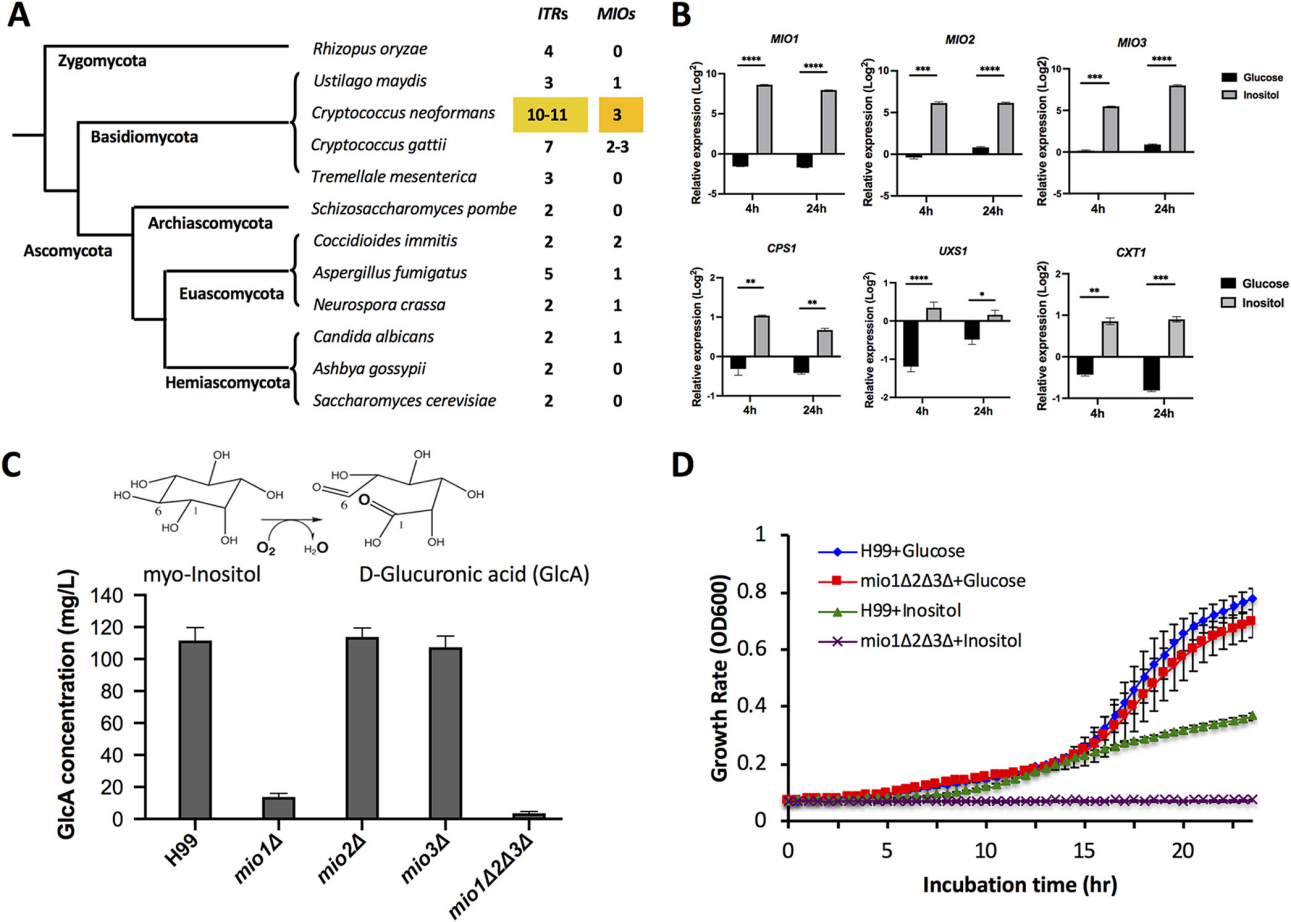
Genes involved in inositol catabolic pathway were highly induced by inositol. (A) Number of *ITR* and *MIO* homologs in different fungal species. (B) Genes involved in the inositol catabolic pathway (*MIO*, *CPS1*, *UXS1*, and *CXT1*) were highly induced by inositol based on qRT-PCR analysis. qRT-PCR was used to quantify the expression changes of the listed genes in wild-type H99 incubated for 4 h or 24 h in medium containing 1% inositol or 1% glucose compared to YPD culture. The relative expression changes were calculated using the ΔΔ*C_T_* method. Statistical analysis was done using Student's *t* test. ****, *P* < 0.0001; ***, *P* < 0.001; **, *P* < 0.01; *, *P* < 0.05. (C) *MIO* functions as an inositol oxygenase in C. neoformans. The oxidation reaction to convert *myo*-inositol to d-glucuronic acid is catalyzed by *myo*-inositol oxygenase. Mio enzyme activity was analyzed by measuring d-glucuronic acid production in H99 and its *mio1Δ*, *mio2Δ*, and *mio3Δ* single mutants and a *mio1Δ2Δ3Δ* triple mutant. (D) Growth rates of wild-type H99 and its *mio* mutants on YNB liquid medium containing either 1% glucose or 1% inositol. The *mio1*Δ*2*Δ*3*Δ triple mutant failed to grow on YNB with inositol as the sole carbon source.

Next, we sought to determine the genes responsible for the enhanced virulence observed in mouse infections. As noted above, C. neoformans expresses 10 or 11 ITRs. Interestingly, this clade also encodes three *myo*-inositol oxygenase (MIO)-encoding genes (*MIO1*, *MIO2*, and *MIO3*) (Fig. 2A; Fig. S2A). Analysis of the gene expression profiles for yeast cells cultured under different inositol conditions shows that genes involved in the putative inositol catabolic pathway are highly upregulated following inositol treatment (18) (Fig. 2B). Quantitative reverse transcription-PCR (qRT-PCR) data show upregulation of all three MIO genes (*MIO1*, *MIO2*, and *MIO3*) in response to inositol, with a >50-fold change compared to their expression in glucose containing medium after 4 or 24 h. While this observation was not entirely unexpected, because C. neoformans can utilize inositol as a sole carbon source, we sought to further understand the role of this pathway in virulence.

10.1128/mBio.02790-21.2 FIG S2 C. neoformans contains three highly conserved *MIO* genes that encode *myo*-inositol oxygenase. (A) Alignment of amino acid sequences of Mio homologs. (B) All three MIO genes are expressed in C. neoformans based on the PCR analysis of genomic DNA and cDNA templates. (C) Growth assay of wild type H99 and its *mio* mutants on YPD and YNB agar plates containing 1% glucose or 1% inositol as a sole carbon source, or under different stress conditions (0.03% SDS, Congo red, and calcofluor white). The *mio1*Δ*2*Δ*3*Δ triple mutant failed to grow on YNB with inositol as a sole carbon source. Cells were incubated on YPD or YNB media and incubated for 48 h before photography. Download FIG S2, TIF file, 1.4 MB. Copyright © 2021 Wang et al. 2021 Wang et al. https://creativecommons.org/licenses/by/4.0/ This content is distributed under the terms of the Creative Commons Attribution 4.0 International license.

MIO oxidizes *myo*-inositol into d-glucuronic acid in the first step of the inositol catabolic pathway. To determine the role of MIO genes in inositol catabolism during fungal development and virulence, we first confirmed that all three genes were expressed and that none of them were pseudogenes, as a previous report suggested (19) (Fig. S2B). Then, to confirm the function of these *MIO* genes, we generated single mutants of each MIO as well as the *mio1*Δ*2*Δ*3*Δ triple mutant and assayed these mutant strains for the production of glucuronic acid using a d-glucuronic acid assay kit (Megazyme, Ireland). Our data showed that while the level of d-glucuronic acid production in the *mio2Δ* and *mio3Δ* single mutants was similar to that of the wild-type strain H99, its production in the *mio1Δ* single mutant was significantly reduced, suggesting that Mio1 is the main protein in this metabolic pathway and plays a predominant role in inositol oxidation. The *mio1Δ2Δ3Δ* triple mutant completely blocked d-glucuronic acid production, indicating that the mutant lacks inositol oxidation activity (Fig. 2C). Interestingly, both the *mio3*Δ and the *mio1Δ2Δ3Δ* mutants showed a modest growth defect on medium containing SDS or Congo red, indicating a minor fitness defect (Fig. S2C). We repeated the assay with two independent mutant isolates from opposite mating types for each mutant, with similar outcomes. These measurements suggest that, indeed, the *MIO* genes encode active inositol oxygenases with functional redundancy.

### *MIO* genes are required for inositol-induced capsule growth.

To understand the role of the inositol catabolic pathway in fungal development and fungal virulence, we examined the phenotype of *mio* mutants in *in vitro* culture conditions. Because inositol can be used as a sole carbon source for C. neoformans, we started by measuring the growth rate of the *mio* mutants in medium with either glucose or inositol as a carbon source. Since complementation strains of the triple mutant proved challenging to create, we used two independent *mio1*Δ*2*Δ*3*Δ triple mutant isolates from opposite mating types, as well as a partially complemented mutant expressing the *MIO1* gene. In agar and liquid growth measurements, the triple mutants showed growth comparable to that of wild-type H99 in medium containing glucose (YPD and yeast nitrogen base [YNB]) but no growth in medium with inositol as the sole carbon source (Fig. 2D; Fig. S2C). These data demonstrate that Mio proteins are essential for C. neoformans to utilize inositol as a carbon source, consistent with their function as an inositol oxygenase.

**FIG 3:**
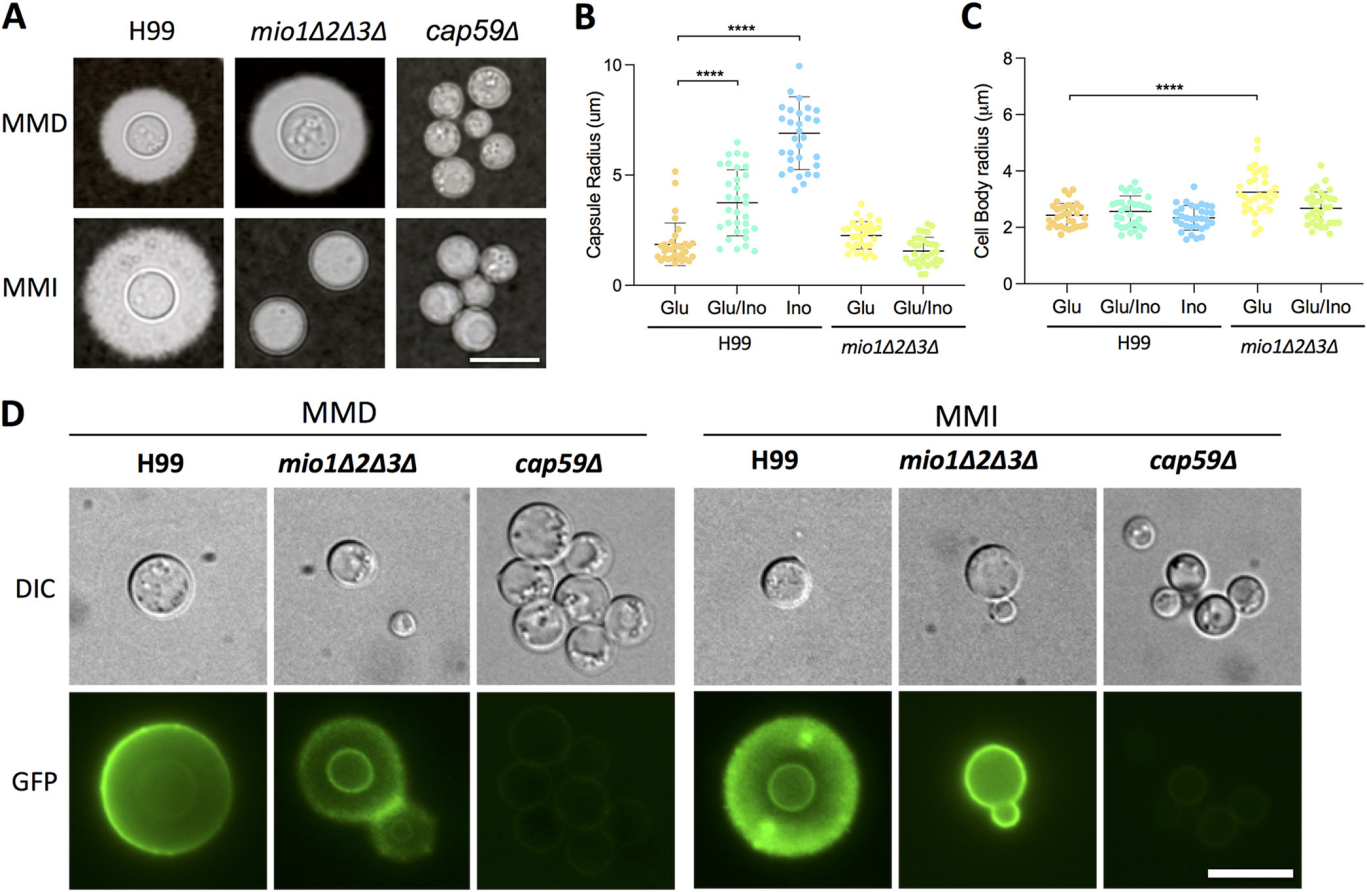
*MIO* genes are required for capsule growth under inositol-inducible conditions. (A) Wild type H99 and its *mio1Δ2Δ3Δ* triple mutant were incubated on capsule-inducing minimal medium with glucose (MMD) or inositol (MMI) as the carbon source for 3 days before photography. (B and C) Capsule size and cell body size of H99 and its *mio1Δ2Δ3Δ* mutants cultured in glucose (Glu), inositol (Ino), or both (Glu/Ino) were analyzed by measuring over 100 cells. Statistical analysis was done using two-tailed Student's *t* test. ****, *P* < 0.0001. (D) Detection of capsule using FITC-labeled GXM monoclonal antibody 18B7. Wild-type H99 and its *mio1Δ2Δ3Δ* mutants were cultured on MM for 3 days before coincubation with the GXM monoclonal antibody 18B7. The acapsular *cap59*Δ mutant was used as a control. Representative images are shown. Bar, 10 μm.

Because larger capsule sizes were observed when C. neoformans strain H99 was cultured under inositol-rich conditions (Fig. 1), we sought to define the role of inositol in capsule growth. The modified minimal medium containing 0.3% glucose (MMD) has been commonly utilized to induce capsule growth in C. neoformans (20). Our data showed that when H99 was cultured on MM containing 0.3% inositol as the carbon source (MMI), it produced much larger capsules compared to culturing on glucose-containing medium (MMD) (Fig. 3A). An inositol dose response analysis showed that the minimal concentration required for inducing capsule growth was ∼166.5 μM (Fig. S3). Since we observed that inositol can induce capsule growth, we also examined how the *mio1*Δ*2*Δ*3*Δ triple mutation affected the capsule production. We found that the capsule of the *mio1*Δ*2*Δ*3*Δ triple mutant was significantly reduced when this strain was cultured with inositol as a carbon source (Fig. 3B). Interestingly, we also observed that the *mio1Δ2Δ3Δ* triple mutant produced significantly larger yeasts but not capsule sizes when cultured with glucose, suggesting that inositol oxygenase enzymes may have additional roles other than the production of glucuronic acid (Fig. 3C).

10.1128/mBio.02790-21.3 FIG S3 Inositol dose response analysis. An inositol dose response analysis of wild-type H99 strain was performed in YNB liquid medium containing different concentration of inositol as the carbon source. It showed that the minimal concentration required for inducing capsule growth was ∼166.5 μM. Download FIG S3, TIF file, 0.5 MB. Copyright © 2021 Wang et al. 2021 Wang et al. https://creativecommons.org/licenses/by/4.0/ This content is distributed under the terms of the Creative Commons Attribution 4.0 International license.

To further characterize the capsules in the *mio1*Δ*2*Δ*3*Δ triple mutant, we performed immunofluorescence (IF) assays using fluorescein isothiocyanate (FITC)-labeled glucuronoxylomannan (GXM) monoclonal antibody (MAb) 18B7. While H99 and the *mio1*Δ*2*Δ*3*Δ triple mutant cultured with glucose looked similar, they varied greatly when cultured with inositol. While the H99 capsule was very large, the *mio1*Δ*2*Δ*3*Δ triple mutant showed a signal only at the cell wall (Fig. 3D), indicating that although there was no capsule growth, the capsule structure was not completely abolished in the triple mutant. This was confirmed using the acapsular *cap59*Δ mutant, which showed no 18B7 fluorescence staining. One interesting observation comes from the immunofluorescence assays. When H99 was grown in inositol medium, the fluorescence was uneven with a punctal pattern, unlike when it is grown in glucose medium, where the fluorescent signal is mostly homogenous and annular. This suggests differences in the capsular architecture induced by a change in carbon source to inositol (Fig. 3D).

### Inositol both induces capsule growth and alters capsule structure.

**FIG 4:**
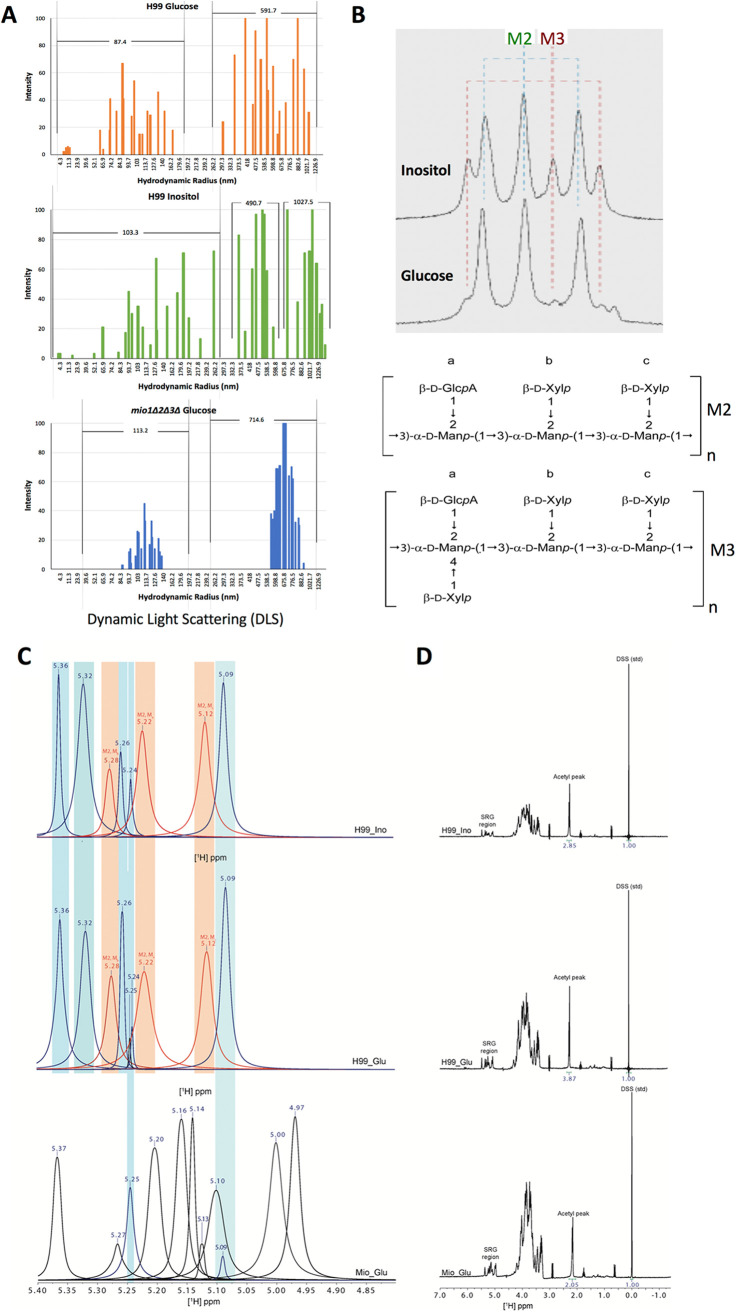
The inositol catabolic pathway effects overall polysaccharide capsular structure. (A) Dynamic light scattering (DLS) reveals distinct polysaccharide characteristics in H99 and its *mio1Δ2Δ3Δ* mutant cultured with glucose or inositol as the carbon source. Three samples of extracellular polysaccharide (EPS) were examined by DLS; H99 cultured in glucose (top) or in inositol (middle) and the *mio1Δ2Δ3Δ* mutant cultured in glucose (bottom). Data for two biological replicates were averaged. While *mio1Δ2Δ3Δ* and H99 cultured in glucose show two size populations, H99 cultured in inositol shows three size populations, one larger than the others. (B) 1D-NMR analysis of GXM structure for samples isolated using the CTAB method from H99 culture in YPD medium with 1% glucose or 1% inositol as the carbon source. The SRG region peaks are shown. Peaks indicating M2 and M3 mannose triad structure are labeled. The basal molecular structure units of M2 and M3 structure are shown at the bottom. (C) Native-form GXM from H99 grown in glucose or inositol shows similar SRG peaks, and both contain the M2 motif of GXM. However, the *mio1Δ2Δ3Δ* triple mutant shows a completely different SRG peak set and does not contain the M2 motif. (D) Analysis of the full ^1^H 1D-NMR spectrum shows that the acetylation of the polysaccharides differs. Each acetyl peak was integrated and compared to the internally consistent D6 DSS standard. While H99 grown in glucose shows a peak area of 3.87 Hz compared to the DSS control, H99 grown in inositol shows decreased acetylation at a peak area of 2.85 Hz, while the *mio1Δ2Δ3Δ* triple mutant shows the smallest acetylation peak area at 2.05 Hz.

To better understand the inositol induced capsule change, we examined the shed polysaccharide, or exopolysaccharide (EPS), through biochemical, biophysical, and immunological means. First, we examined the size of EPS polymers using dynamic light scattering (DLS) (Fig. 4A). We observed two size populations of EPS from C. neoformans grown in glucose medium (average hydrodynamic radii of 87.4 nm and 591.7 nm) but three size populations when grown in inositol medium (average hydrodynamic radii of 103.3 nm, 490.7 nm, and 1,027.5 nm). The largest polymers were obtained from C. neoformans grown in inositol-containing minimal medium (MMI), at 1,226.9 nm (Fig. 4A).

Next, we examined the EPS using resolution-enhanced one-dimensional nuclear magnetic resonance (1D-NMR). Utilizing this technique, Cherniak et al. previously reported that the chemical shifts of the anomeric mannose residues could be used to define the different motifs of GXM, referred to as structural reporter groups (SRGs) (21). For this analysis, we utilized both cetyltrimethylammonium bromide (CTAB)-extracted EPS and native EPS. The CTAB-extracted EPS spectrum from cells grown on glucose medium showed three major mannosyl anomeric peaks at 5.294, 5.240, and 5.169 ppm (Fig. 4B) (Table 1), consistent with the SRG for the M2 mannosyl triad. However, a minor peak pattern comprising three signals at 5.316, 5.202, and 5.142 ppm was also present; this peak set was consistent with the M3 triad. In the sample isolated from inositol medium, peak sets of the M2 and M3 triads were both present, but the M3 peak set was more prominent in the inositol sample than the glucose sample, suggesting an increase in the M3 motif (Fig. 4B). However, analysis of the native EPS did not replicate these results. 1D-NMR analysis of native EPS shows that the peak sets from the inositol- and glucose-cultured C. neoformans vary little; both contain the M2 mannosyl triad (Fig. 4C). Interestingly, the 1D-NMR showed differences in global O-acetylation between the two culture conditions. Glucose-cultured C. neoformans had an acetyl peak area of 3.87 Hz (relative to the 2,2-dimethyl-2-silapentane-5-sulfonic acid [DSS] internal control), while the acetyl peak in inositol-cultured C. neoformans was smaller, 2.85 Hz, indicating reduced O-acetylation in inositol conditions (Fig. 4D). The CTAB method of EPS isolation alters the polysaccharides, reducing their size and complexity (22). While the native EPS structure was not altered in any way, this may mask changes in the polymer that are exposed only by the CTAB method. Consistent through both methods of analysis was the observed M2 triad and changes in the polysaccharide O-acetylation pattern induced by inositol.

**TABLE 1:** Line fitting results of the two EPS spectra and calculated percentage of M2 and M3 mannosyl triads

H99 carbon source	Peak signal (ppm)	Area	Triad	Avg area	Area %
Glucose					
M2a	5.240	1,370.61			
M2b	5.294	1,125.46			
M2c	5.169	1,146.88	M2	1,214.317	88

M3a	5.202	216.35			
M3b	5.316	195.39			
M3c	5.142	95.77	M3	169.1713	12

Inositol					
M2a	5.242	2,737.13			
M2b	5.289	2,592.29			
M2c	5.172	2,385.12	M2	2,571.514	70

M3a	5.204	1,215.50			
M3b	5.312	1,131.56			
M3c	5.145	1,010.95	M3	1,119.338	30

### Loss of inositol oxygenase changes global polysaccharide structure.

To understand how the *MIO* genes contribute to the fungal capsule size and structure, we further analyzed the GXM structure produced by H99 and the *mio1*Δ*2*Δ*3*Δ triple mutants when grown in medium with different carbon sources. Because glucose and inositol showed differences in EPS, we utilized 1D-NMR to also compare the GXM structure in wild-type H99 and its *mio1Δ2Δ3Δ* mutants. As noted above, CTAB EPS isolation can alter the structure of polysaccharides (23), so for this analysis we utilized native EPS. We detected a particle size difference in the polysaccharide secreted by strains grown on media with different carbon sources (Fig. 4A). 1D-NMR shows that there is no significant difference between native EPS SRGs from H99 cultured in glucose and those from H99 grown in inositol. However, EPS from the *mio1Δ2Δ3Δ* mutant showed significant differences in the SRG peak set (Fig. 4C). The SRG peaks observed in the *mio1Δ2Δ3Δ* mutant do not correlate with the M2 motif found in the glucose- and inositol-cultured wild-type strains or any of the characterized GXM motifs. Additionally, EPS from the *mio1Δ2Δ3Δ* mutant showed decreased acetylation (peak area, 2.05). Taken together, these observations suggest that the *mio1Δ2Δ3Δ* mutation results in global changes to EPS and perhaps in new GXM motifs.

Overall, our data show that inositol feeding leads to global changes in capsule growth and polysaccharide structure in C. neoformans that affects both the capsule size and its secretion. Yeast cells grown on inositol medium secrete larger polysaccharides that are distinct from the characterized GXM motifs and have altered O-acetylation patterns.

### Inositol catabolism is required for fungal virulence.

**FIG 5:**
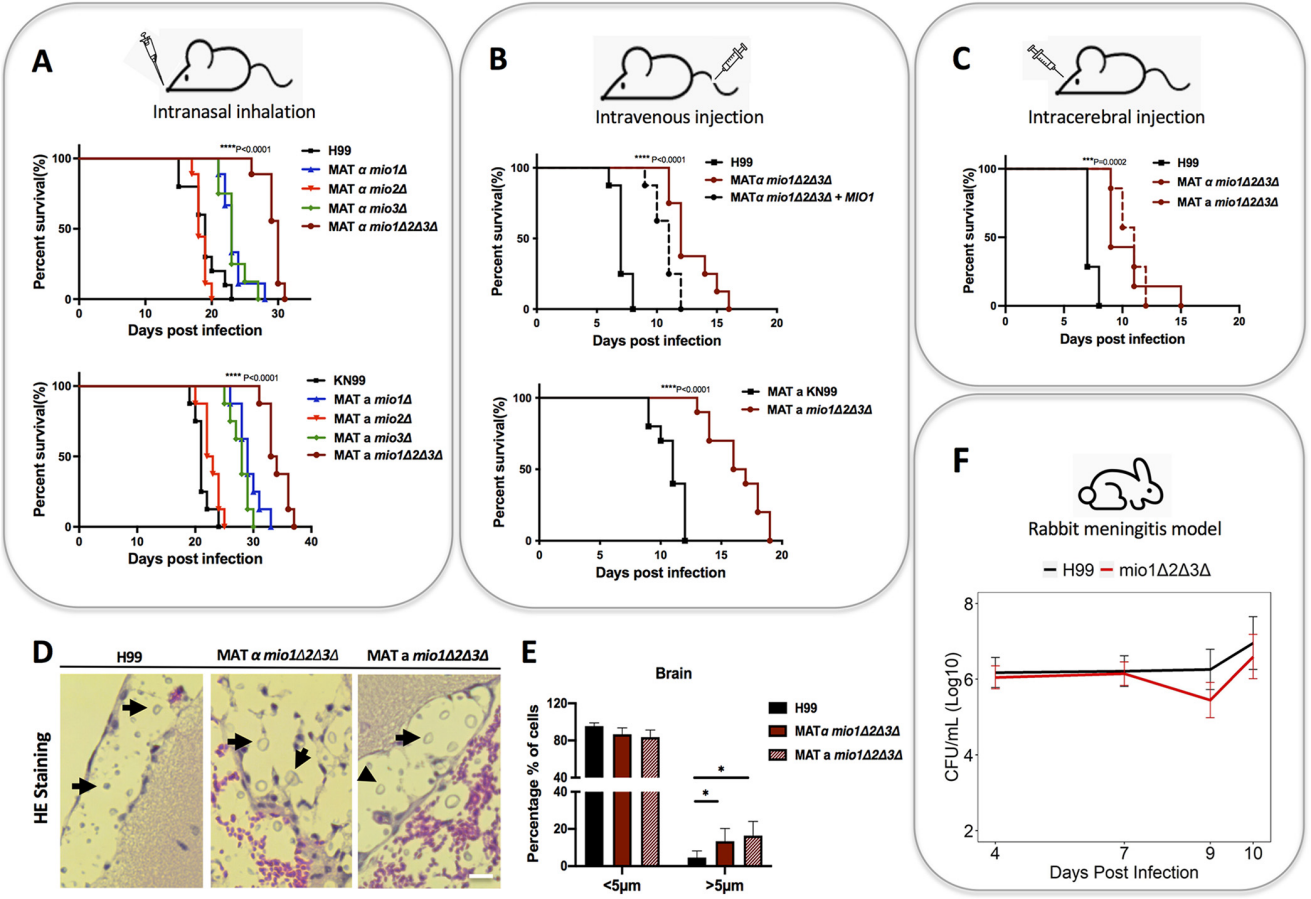
*MIO* genes are required for fungal virulence in animal models of systemic cryptococcosis. (A) Survival rates of wild type H99 and the *mio1*Δ, *mio2*Δ, *mio3*Δ, and *mio1*Δ*2*Δ*3*Δ mutants in a murine intranasal inhalation infection model of systemic cryptococcosis. Strains of both mating types of each mutant and its parental wild type were tested in the same infection model to make sure independent isolates of the same mutant were used in the animal study. ****, *P* < 0.0001; statistical analysis was done using a log rank (Mantel-Cox) test. (B) Survival rates of H99 and its *mio1*Δ*2*Δ*3*Δ mutants in a murine intravenous injection infection model of systemic cryptococcosis. Both mating types of the mutant and its parental wild type, as well as a partially complemented strain (*mio1Δ2Δ3Δ MIO1*), were tested in the same infection model. ****, *P* < 0.0001; statistical analysis was done using a log rank (Mantel-Cox) test. (C) Survival rates of H99 and its *mio1*Δ*2*Δ*3*Δ mutants of both mating types in a murine intracerebral injection model of cryptococcosis. ***, *P* < 0.001; statistical analysis was done using a log rank (Mantel-Cox) test. (D) H&E staining of mouse brain sections infected with H99 or the *mio1Δ2Δ3Δ* mutants at the endpoint of the intracerebral infection experiment. Yeast cells are highlighted (black arrow). Bar, 10 μm. (E) Quantitative results of yeast cell body size in the infection site in the brain *in vivo*. Proportions of cells smaller or larger than 5 μm in diameter are shown. *, *P* < 0.05 (Student's *t* test). Quantitative cell size data from over 100 cells from each sample are presented. (F) Fungal burden in infected rabbit cerebrospinal fluid at 4, 7, and 9 or 10 days postinjection in a rabbit meningitis model. The data points are the log-transformed mean values on days 4 (H99, *n* = 6; *mio1Δ2Δ3Δ*, *n* = 9), 7 (H99, *n* = 6; *mio1Δ2Δ3Δ*, *n* = 9), 9 (H99, *n* = 3; *mio1Δ2Δ3Δ*, *n* = 2), and 10 (H99, *n* = 2; *mio1Δ2Δ3Δ*, *n* = 3) postinfection. Error bars show log-transformed standard errors of the means (SEM).

Capsule production is an essential virulence factor in C. neoformans. Due to the observed involvement of the inositol catabolic pathway in capsule size and structure, we were curious about the potential contribution of MIOs to fungal virulence. To test this possibility, we used multiple infection models in mice, including intranasal inhalation, intravenous injection, and intracerebral injection methods (Fig. 5). We performed virulence assays in mice with *mio1Δ*, *mio2Δ*, and *mio3Δ* single mutants as well as *mio1Δ2Δ3Δ* triple mutants from both mating type backgrounds as independent mutant isolates. Our mouse survival studies demonstrated that both *mio1Δ* and *mio3Δ* mutations but not the *mio2Δ* mutation resulted in significant virulence attenuation (Fig. 5A). The *mio1Δ2Δ3Δ* mutant also showed significant virulence attenuation in all three murine models of cryptococcosis (Fig. 5A to C). This virulence attenuation is incompletely rescued in the partially complemented *mio1Δ2Δ3Δ MIO1* mutant (Fig. 5B). The significant virulence attenuation in the *mio1Δ* and *mio1Δ2Δ3Δ* mutants agrees with our previous observation that Mio1 is the protein predominantly responsible for the inositol oxygenase activity observed in wild-type strains. The virulence attenuation in the *mio3Δ* mutant was not expected, as this mutant did not show a defect in d-glucuronic acid production. It is possible that Mio3 protein has additional functions that remain to be elucidated. Histopathology of infected mouse brain tissue sections indicates that, in general, the *mio1*Δ*2*Δ*3*Δ triple mutant cells are larger than wild-type cells (Fig. 5D and E). This is consistent with our *in vitro* analysis, in which the wild-type H99 cells were significantly smaller than the *mio1*Δ*2*Δ*3*Δ cells when the strains were grown under glucose conditions (Fig. 3C).

To confirm these results in another animal model, we examined the effects of the *mio1Δ2Δ3Δ* triple mutation in an immunosuppressed rabbit cryptococcal meningitis model. In this model, the yeasts must survive in the clinically relevant subarachnoid space. We did not observe a significant reduction in fungal CFU burden in rabbits infected with the same inoculum of the *mio1*Δ*2*Δ*3*Δ triple mutant compared to those receiving wild-type H99 (Fig. 5F). The subarachnoid space is continuously supplied with both inositol and glucose, which might explain the lack of change in fungal burden. During the later stages of infection (days 9 to 12), we began to observe a CFU reduction, suggesting a modest attenuation in the *mio1*Δ*2*Δ*3*Δ triple mutant, but this did not reach statistical significance. Together, these results indicate that inositol catabolism is likely necessary for optimal virulence but its impact on yeast survival in the host is nuanced, depending on site of infection, length of infection, animal model, and the read-out endpoints of disease.

In aggregate, these results show that the inositol catabolic pathway not only provides C. neoformans cells with energy by catabolizing free inositol but also supplies yeast cells with d-glucuronic acid, a key substrate for its major virulence factor, the polysaccharide capsule. The inositol-mediated changes in the polysaccharide capsule have an impact on fungal virulence by affecting the production of capsule, its growth, and its structure. The nuances of how these inositol-mediated capsular changes play out during infection as well as the role of inositol as a signaling molecule and how fungal cells sense inositol require further study.

## DISCUSSION

The Cryptococcus capsule is a well characterized virulence factor that is composed of two major polysaccharides, glucuronoxylomannan (GXM), which accounts for 90% of the capsular polysaccharide (CPS), and galactoxylomannan (GalXM), which accounts for approximately 10% (24, 25). GXM is composed of an α-(1,3)-mannose backbone decorated with variable glucuronic acid and xylose side chain branches (24, 26). Glucose, a commonly used carbon source in studying capsule formation, is converted into UDP-glucuronic acid (GlcA) by UDP-glucose dehydrogenase (Ugd1) (27), which is further converted into UDP-xylose (Xyl) by UDP-glucuronic acid decarboxylase (Uxs1) (28) to produce two of the building blocks of GXM. Following a similar series of events, fructose-6-phosphate is converted into GDP-mannose (29), yielding the final component of GXM. While the building blocks of GXM are GDP-mannose (Man), UDP-GlcA, and UDP-Xyl, how these components are put together is not well understood.

While glucose is the most commonly used carbon source in the study of C. neoformans and its capsule, most environmental niches for Cryptococcus contain multiple carbon sources, including inositol. In fact, C. neoformans can use inositol as a sole carbon source, and recent analysis shows that the inositol metabolic pathway remains active even under high-glucose conditions (13). When inositol is the only carbon source, cryptococcal cells still produce large capsules, suggesting that inositol may also be utilized as a substrate for capsule production. Consistent with this notion, the *MIO* genes, encoding *myo*-inositol oxygenase, which converts inositol into d-GlcA, are upregulated under inositol growth conditions (Fig. 2B). Also upregulated under inositol growth conditions (Fig. 2B), UDP-glucuronate decarboxylase, encoded by UXS1, converts UDP-GlcA to UDP-Xyl (28). When the *MIO* genes are deleted in the *mio1Δ2*Δ*3*Δ triple mutant, little or no GlcA is produced (Fig. 2C), indicating that these *MIO* genes are necessary to produce GlcA for capsular polysaccharide production under inositol growth conditions.

Analysis of capsules produced by either the wild type or the *mio1*Δ*2*Δ*3*Δ triple mutants revealed striking alterations. Based on the seminal work by Cherniak et al. in the 1990s (30), structural analysis of C. neoformans polysaccharides generally relies on the anomeric proton chemical shifts of each backbone mannose residue, as determined by ^1^H 1D-NMR. Because the chemical shifts of anomeric protons in polysaccharides are highly sensitive to neighbor effects, they are often referred to as structural reporter groups (SRGs) and are useful in defining primary sequence motifs of GXM. Additionally, the 6-O-acetylation (6-OAc) of the mannose residues is critical for antibody binding, suggesting that this chemical modification is important to secondary and tertiary confirmations of GXM epitopes. Since 6-OAc modification is not factored into the SRG analysis, we extended the SRG analysis to more fully define GXM structure. Utilizing the CTAB methods developed by Cherniak, we observed that the EPS of wild-type strains grown in glucose predominantly contains the M2 motif of GXM (Fig. 4B), with minor M3 motif peaks. When the wild-type strain is grown in inositol medium, however, the peak set is altered and includes greater-intensity peaks, consistent with the M3 motif. The difference between the M2 and M3 motifs is the addition of a β(1,4)-linked xylose in the M3 motif, consistent with increased GlcA and Xyl production under inositol conditions. These observations were not apparent in native EPS prepared by filtration. This suggests that the processing method involving CTAB precipitation and lyophilization changes the EPS, perhaps revealing motifs that are otherwise unavailable due to secondary and tertiary confirmations.

To further our understanding of changes in the polysaccharides, we examined both the SRG region and 6-OAc of the EPS from a wild-type strain grown in inositol and the *mio1*Δ*2*Δ*3*Δ triple mutant and observed global changes to the natively prepared polysaccharides. The *mio1*Δ*2*Δ*3*Δ triple mutant SRG region showed no peak sets in common with characterized GXM motifs (Fig. 4C). This is not surprising given that production of GlcA is lost in the *mio1*Δ*2*Δ*3*Δ triple mutant and that each GXM motif is reported to contain a GlcA for every trimannose repeat. Additionally, we observed changes in 6-OAc of the mannose backbone for both the wild type and the *mio1*Δ*2*Δ*3*Δ triple mutant grown under inositol conditions. This is surprising given that only GlcA and Xyl availability is altered and that Man is the residue which is 6-OAc. It has been suggested that the GlcA branch precedes the 6-OAc of Man, so perhaps the lack of available GlcA also restricts Man 6-OAc. Taken together, these data indicate the complexity of the polysaccharides in C. neoformans and the infancy of our understanding of this complex polysaccharide. More detailed studies are necessary to create a full picture of the specific differences in capsule structure when C. neoformans is grown in different carbon sources.

The molecular effects of inositol metabolism on the cryptococcal capsule also have physiological consequences in fungal virulence. We found that inositol not only induces capsule size but also increases the virulence of C. neoformans in a mouse model. C. neoformans harvested from infected brains showed increased virulence beyond that of the parent strain or yeast harvested from the lungs of infected mice. Both *in vivo* and *in vitro* data on capsule measurement indicate that fungal passages under inositol-rich conditions result in a heritable change in capsule size that may also result in structural changes. Further studies are needed to characterize such a change in detail. Additionally, we observed that the deletion of *MIO* genes decreased virulence compared to the wild-type strain in murine infection models (Fig. 5A to C). This indicates that the activity of inositol oxygenases is required for full virulence in C. neoformans (Fig. 2C). The virulence difference between wild type and the *mio1Δ2Δ3Δ* triple mutant in the rabbit meningitis model was decreased. Here, differences are measured by fungal burden in the CSF at different times postinoculation, so the discrepancy may be related to the higher fungal inoculum (10^8^ cells) used in rabbit infection and the difference in host conditions, including the higher rabbit basal temperature relative to that of mice.

Recently, a large-cohort study was conducted to analyze the RNA-Seq transcriptome profiles of a large number of C. neoformans clinical isolates from the cerebrospinal fluid (CSF) of patients with cryptococcal meningoencephalitis (31). To better understand the potential impact of fungal inositol utilization and metabolism during human cryptococcosis, we also analyzed 11 isolates from this large cohort for the expression of genes involved in inositol metabolism. Our data showed that most genes encoding proteins involved in inositol synthesis (INO1 and INM1), inositol metabolism (PI kinases and phosphatases), and inositol catabolism (MIOs) pathways were highly expressed across all CSF samples compared to the expression of the ACT1 gene, indicating that fungal inositol metabolism is highly active during human brain infection (Fig. S4). Because of the role of inositol in capsule growth, we also analyzed the genes involved in polysaccharide biosynthesis. Our data also showed that genes encoding UXS1, CXT1, and CPS1 are also highly expressed during brain infection. These results are consistent with our animal infection data showing that inositol metabolism is active and contributes to fungal infections.

10.1128/mBio.02790-21.4 FIG S4 Comparison of gene expression among clinical isolates during human meningitis. Heat map of RNA-Seq transcriptome analysis for 41 selected genes involved in inositol biosynthesis, inositol metabolism, inositol catabolism, and polysaccharide biosynthesis in 11 human CSF isolates of C. neoformans. The color corresponds to the normalized log CPM values. ACT1 was used as the positive control. Download FIG S4, TIF file, 0.8 MB. Copyright © 2021 Wang et al. 2021 Wang et al. https://creativecommons.org/licenses/by/4.0/ This content is distributed under the terms of the Creative Commons Attribution 4.0 International license.

Nutrient sensing and utilization play a critical role in pathogen adaptation to their host environment and virulence. For most organisms, the critical nutrient is carbon in the form of sugar; the same is true of C. neoformans, though for different reasons. A unique and critical characteristic of C. neoformans is the polysaccharide capsule. Built even under starvation conditions, this capsule protects the fungus from desiccation, but it also proves to be critical in virulence as well. While it is clear that the polysaccharide capsule is crucial for virulence, the role of carbon sources other than glucose in capsule production has not really been explored. The likely expansion of gene families of inositol oxygenase genes and inositol transporters also suggests a microevolution of inositol utilization to adapt to fungal niches, e.g., inositol-rich environments. Here, we show that inositol in culture increases virulence and that this virulence is further enhanced by growth in the mouse brain, a host environment with abundant inositol. A similar increase in virulence is observed when C. neoformans is incubated with the amoeba Dictyostelium discoideum or Acanthamoeba castellanii (32). The observations with amoebas have led to the hypothesis that amoebas represent an environmental “training ground” for the development of virulence factors (33). A similar mechanism may exist for the sensing and utilization of inositol, since the natural reservoirs for C. neoformans, such as plants, are often rich in inositol. Evolutionary analysis indicates that cryptococcal species have developed complex inositol utilization mechanisms, possibly through coevolution with their natural hosts. This is consistent with the observed expansion of the inositol transporter gene family and inositol oxygenase genes in C. neoformans. In the nonencapsulated pathogen Legionella pneumophila, inositol metabolism is essential for its survival and growth in A. castellanii (34), suggesting that inositol metabolism may contribute to the observed increase in virulence outside its effects on the capsule.

**FIG 6:**
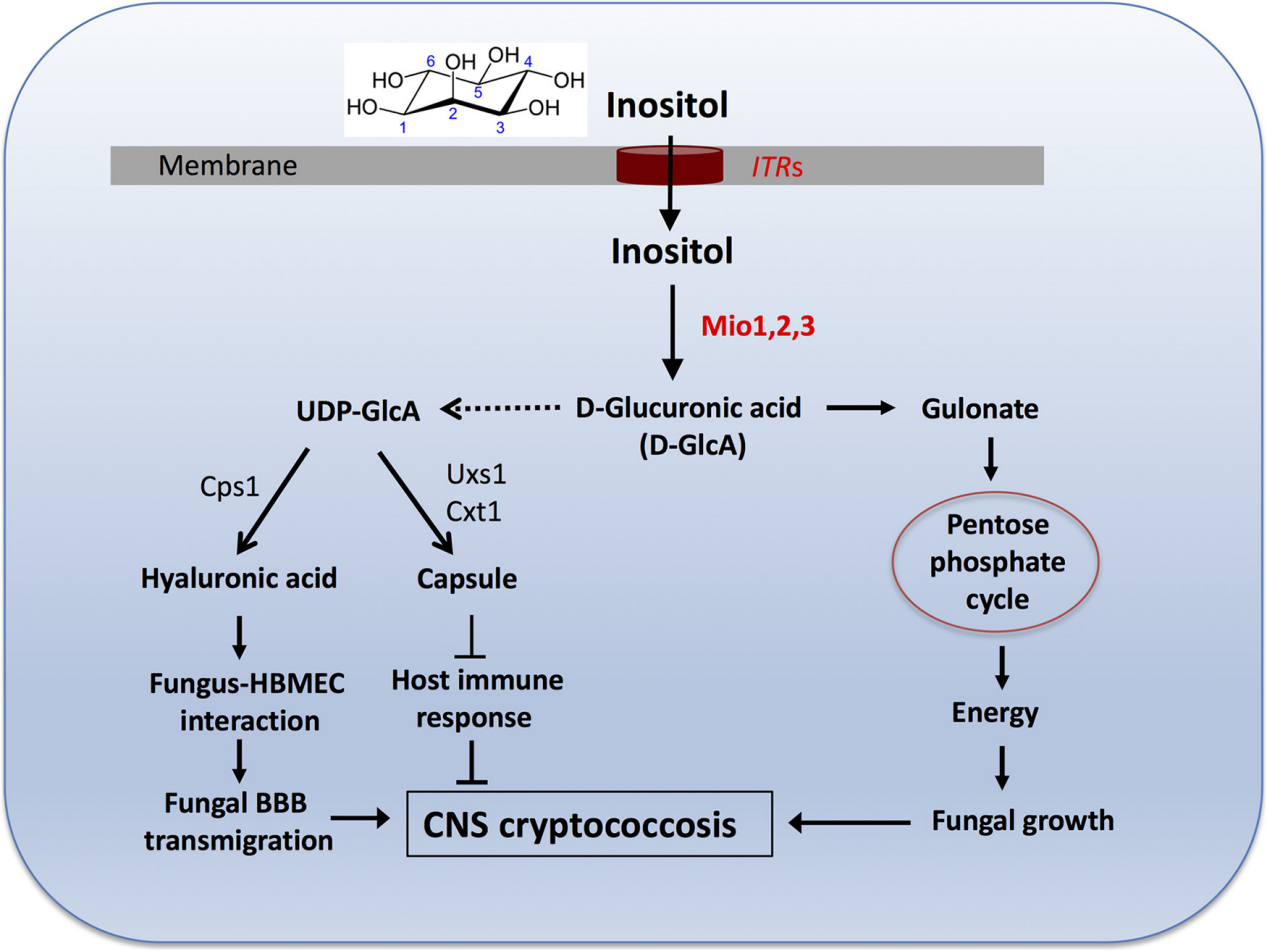
Working model. Extracellular inositol taken up by the inositol transporters ITRs is oxidated by the inositol oxygenase (MIOs) to produce d-glucuronic acid. d-Glucuronic acid can be catabolized to produce gulonate, which can be reduced to the pentose phosphate cycle to provide energy to support fungal growth. d-Glucuronic acid can also provide a necessary substrate to produce UDP-glucuronic acid, which is a substrate for both disaccharide hyaluronic acid (HA) and polysaccharide capsule. HA has been shown to interacts with the host CD44 receptor to induce yeast transmigration across the BBB, while capsule is a major virulence factor that can compromise host protective immunity. Together, inositol-mediated energy support and polysaccharide production promote disease progression during CNS cryptococcosis.

In aggregate, C. neoformans has acquired a unique ability to utilize environmental and host inositol for its growth, replication, and virulence. As a part of the complex inositol sensing and utilization strategy, the inositol oxidation pathway contributes to fungal virulence by supplying both energy and substrate glucuronic acid for the construction of fungal cell surface structures (hyaluronic acid and capsule) (Fig. 6). Hyaluronic acid was reported to interact with the CD44 receptor in human brain macrovascular endothelial cells (HBMEC) to promote C. neoformans transmigration across the blood-brain barrier (BBB) during infection of the central nervous system (CNS) (35, 36). Our previous study found that inositol induces the expression of hyaluronic acid synthase (Cps1) and hyaluronic acid production (13, 37). Taken together with the known importance of inositol transporters (ITRs) (11) and inositol pyrophosphate biosynthetic pathway (38) in fungal development and virulence, this study has led to a better understanding of how the fungal cells take up and utilize inositol in capsule-mediated virulence to promote CNS cryptococcosis. This work also raises several further questions about functional roles for inositol in C. neoformans. Inositol has been reported to function as a signaling molecule (39, 40), but how this function induces Cryptococcus capsule size remains to be studied. A receptor to sense inositol, if there is one, remains unknown and warrants further investigation. The novel contribution of inositol to cryptococcal virulence is a highly exciting area of research that has also been suggested by large-scale investigation of clinical and environmental Cryptococcus isolates and population genetics study (41).

## MATERIALS AND METHODS

### Ethics statement.

All animal studies were conducted following biosafety level 2 (BSL-2) protocols and procedures approved by the Institutional Animal Care and Use Committee (IACUC) and Institutional Biosafety Committee of Rutgers University and Duke University, respectively. The studies were conducted in facilities accredited by the Association for Assessment and Accreditation of Laboratory Animal Care (AAALAC).

### Strains, media, and growth conditions.

C. neoformans strains used in this study are listed in Table 2. Strains were grown at 30°C on yeast extract-peptone-dextrose (YPD) agar medium and synthetic defined (SD) medium. V8 medium (pH 5.0) was used for mating assays. Modified MS medium (Murashige and Skoog medium) was used for mating and sporulation assays and prepared as previously described (10). YNB medium without inositol was purchased from Sigma-Aldrich. Niger seed medium was used to test for melanin production. Dulbecco modified Eagle’s (DME) medium for assessing capsule production was prepared as previously described (10). All other media were prepared as previously described (42–44).

**TABLE 2:** C. neoformans strains used in this study

Strain	Genotype or description	Source or reference
H99	Wild type	J. Perfect
CUX122	*MAT*α *mio1*::*NEO*	This study
CUX123	*MAT***a** *mio1*::*NEO*	This study
CUX124	*MAT*α *mio2*::*NAT*	This study
CUX125	*MAT***a** *mio2*::*NAT*	This study
CUX203	*MAT*α *mio3*::*NEO*	This study
CUX127	*MAT*α *mio1*::*NEO mio2*::*NAT*	This study
CUX128	*MAT***a** *mio1*::*NEO mio2*::*NAT*	This study
CUX204	*MAT*α *mio1*::*NEO mio2*::*NAT mio3*::*NEO*	This study
CUX205	*MAT***a** *mio1*::*NEO mio2*::*NAT mio3*::*NEO*	This study
CUX215	*MAT*α *mio1*::*NEO mio3*::*NEO*	This study
CUX216	*MAT***a** *mio1*::*NEO mio3*::*NEO*	This study
CUX837	*MAT*α *mio1*::*NEO MIO1-NAT*	This study
CUX838	*MAT***a** *mio1*::*NEO MIO1-NAT*	This study
CUX839	*MAT*α *mio1*::*NEO mio2*::*NAT mio3*::*NEO MIO1*:*URA5*	This study
PMH1033_hCSF	Clinical isolate	31
PMH1040_hCSF	Clinical isolate	31
PMH1051_hCSF	Clinical isolate	31
NRH5010_hCSF	Clinical isolate	31
NRH5081_hCSF	Clinical isolate	31
NRH5084_hCSF	Clinical isolate	31
PMH1063_hCSF	Clinical isolate	31
NRH5076_hCSF	Clinical isolate	31
NRH5027_hCSF	Clinical isolate	31
PMH1065_hCSF	Clinical isolate	31
NRH5045_hCSF	Clinical isolate	31

A total of 11 C. neoformans strains, representing three genotype groups, including 4 VNI, 4 VNBI, and 3 VNBII strains, were isolated directly from the cerebrospinal fluid (CSF) from individual patients with advanced HIV infections and low CD4 counts by means of a lumbar puncture as standard of care. Both Duke and Botswana institutional review board (IRB) approvals (PRO00029982) supported this study. RNA was extracted and isolated following the previous protocol (45). The mRNA purification was performed using a TruSeq RNA sample preparation kit following the manufacturer’s guidelines (RNeasy minikit; Qiagen, Valencia, CA). The cDNA libraries were sequenced on Illumina HiSeq 2000 instruments (Illumina, San Diego, CA). The paired-read alignment was carried out against the transcriptomes of C. neoformans var. *grubii* H99 (CNA3) using STAR (v 2.5.3a) (46). Subsequently, the read counts for each gene were estimated with RSEM (v1.2.31) (47). The raw count data were converted to counts per million (CPM) and then normalized by adjusting with the effective library size via the calcNormFactors function implemented in the R package edgeR (v. 3.26.8) (48).

### Detection of *MIO* gene expression using quantitative RT-PCR.

To test how the *MIO* genes respond to the presence of environmental *myo*-inositol, both *in vitro* and *in vivo*, we measured the mRNA levels for *MIO* genes under different conditions via quantitative real-time PCR (qPCR). Cultures of C. neoformans wild-type strain H99 and its mutant strains were grown on YPD for 24 h at 30°C. Collected cells were washed with distilled water (dH_2_O), and pellets were used for total RNA extraction. Total RNAs were extracted using TRIzol reagents (Invitrogen) and purified with an RNeasy cleanup kit (Qiagen) following the manufacturer’s instructions. Purified RNAs were quantified using a Nanodrop instrument (Thermo Scientific).

First-strand cDNAs were synthesized using a Superscript III cDNA synthesis kit (Invitrogen) following the instructions provided by the manufacturer. Expression of *ITRs* and *GAPDH* were analyzed with the comparative cycle threshold (*C_T_*) method using Brilliant SYBR green qPCR reagents (TaKaRa) as described previously (12).

### Generation of *mio* mutants (single mutants and triple mutants).

In mating assays, C. neoformans cells of opposite mating types were mixed and cocultured on V8 or MS agar medium at 25°C in the dark for 10 days and filamentation was examined by light microscopy. Spore production was visualized by microscopy and photographed. Basidiospores were dissected from mating assays performed on MS medium.

To generate *mio1 mio2* (*mio1*Δ*2*Δ) double mutants, a mating between *MAT*α *mio1*::*NEO* and *MAT***a** *mio2*::*NAT* mutants was conducted, and spores were visualized and isolated with an MSM system (Singer Instruments, England). Genomic DNA was isolated from all progeny that grew on YPD with both nourseothricin and G418. PCR was used to screen for *MIO1* gene deletion with primers CX270/JH8994 and CX268/CX269, and for *MIO2* gene deletion with primers CX277/JH8994 and CX262/CX263 (Table S1). To generate *mio1*Δ*2*Δ*3*Δ triple mutants, a mating between a *MAT*α *mio1*::*NEO mio2*::*NAT* double mutant and a *MAT***a** *mio3*::*NEO* mutant was conducted, and spores were isolated. Triple mutants were screened first by PCR on cultures initiated from single spores. All mutants confirmed by PCR were further confirmed by Southern blotting analyses. The mating type of each confirmed mutant strain was determined by PCR using mating type-specific primers and by genetic crossing.

10.1128/mBio.02790-21.5 TABLE S1 Primers used in this study. Download Table S1, DOCX file, 0.02 MB. Copyright © 2021 Wang et al. 2021 Wang et al. https://creativecommons.org/licenses/by/4.0/ This content is distributed under the terms of the Creative Commons Attribution 4.0 International license.

To generate complemented strains of the *mio1*Δ*2*Δ*3*Δ triple mutant, a genomic DNA fragment that contained a 1.5-kb upstream region of the *MIO1* open reading frame (ORF) and its 500-bp downstream region was amplified by PCR with primers CX672/CX673. The *MIO1* PCR fragment was inserted into vector pJAF13 using in-fusion cloning. The construct pJAF13-*MIO1* was biolistically transformed in *mio1*Δ*2*Δ*3*Δ mutant strains. The selected colonies grown on YNB with 1% inositol plates were further confirmed by PCR.

### Assays for capsule size and GXM secretion.

To examine capsule production, yeast cells were grown in YPD broth overnight at 30°C with constant agitation, washed 3 times with phosphate-buffered saline (PBS), and resuspended in modified minimum medium or inoculated on Dulbecco modified Eagle (DME) agar medium. The yeasts in liquid media were grown at 37°C for 5 days and the DME medium plates were incubated at 37°C for 3 days before capsule size was visualized by adding a drop of India ink to the cell suspensions and observing them on an Olympus AX70 microscope (Melville, NY). The relative capsule size was calculated by dividing capsule size by the whole-cell size (capsule and cell size). The average and standard deviation from at least 50 cells were calculated for each condition tested. Secreted total polysaccharides were purified from a 500-ml YPD culture of each strain using the cetyltrimethylammonium bromide (CTAB) precipitation method as described previously (49). The amount of total GXM was determined by the phenol sulfuric method. The two-pair *t* test method was used to determine the statistical significance of the difference between samples.

### Measurement of d-glucuronic acid production.

C. neoformans overnight cultures were prepared in YPD, and cell pellets were resuspended in 5 ml of fresh YNB plus 1% glucose or YNB plus 1% inositol liquid culture overnight at 30°C with shaking. Cells were washed twice with double-distilled water (ddH_2_O), placed at −80°C for 2 h, and lyophilized overnight. Cells were resuspended into 0.6 ml of morpholineethanesulfonic acid (MES) buffer, and suspensions were transferred to 2-ml screw cap tubes containing 0.6 ml glass beads for bead beating for 10 min. Then, the sample was boiled for 10 min to kill all enzyme activities and denature proteins. Cell suspensions were centrifuged for 5 min at high speed. Supernatants were then transferred and kept on ice. Glucuronic acid levels were measured by following the instruction of manufacturer manual (Megazyme, Ireland).

### Isolation of total and fractionated native-form exopolysaccharide from C. neoformans.

Yeast strains were inoculated into Sabouraud broth medium and incubated at 30°C with shaking for approximately 24 h. The cells were washed twice in PBS, and approximately 10^6^ cells/ml were inoculated into modified minimum medium with glucose (7.5 mM), glucose and inositol (3.75 mM each) or inositol (7.5 mM), MgSO_4_ (10 mM), KH_2_PO_4_ (29.4 mM), glycine (13 mM), and thiamine-HCl (3 *μ*M), pH 5.5. The yeast cultures were incubated at 30°C for approximately 72 h with shaking.

Yeast cells were centrifuged at 4,000 rpm for 30 min. The supernatant was collected and filtered through a 0.45-μm Steritop filter. Whole EPS was concentrated by limited lyophilization to a 10× concentration, and the dry weight of the final solution was measured after lyophilization (100 μl dried in weighed tubes). The samples were kept at 4°C until further analysis. To obtain fractionated EPS, the filtered supernatant was passed through filters with molecular weight cutoffs of 100, 10, and 3 kDa.

### Isolation of capsular polysaccharide from C. neoformans.

The capsular polysaccharide was isolated by a protocol modified from reference 50. Briefly, C. neoformans cells were pelleted and washed twice with ultrapure distilled water. Dimethyl sulfoxide (DMSO) was added to a pellet of yeast cells in a ratio of 3:1 by volume, vortexed, and incubated at 30°C with shaking for 1 h. The cells were centrifuged for 1 h at 4,000 rpm, and the supernatant was collected and dialyzed for 7 days using Slide-A-Lyzer dialysis cassettes with a 3,500-Da molecular weight cutoff against ultrapure distilled water. The dialyzed capsular polysaccharide was frozen, lyophilized, and resolubilized in ultrapure distilled water to obtain the desired concentration.

### Total capsule purification using the CTAB method for ^1^H 1D-NMR structure identification.

The yeast samples grown on glucose or inositol medium were pelleted and GXM from the supernatants was purified using the reported CTAB protocol (49). A polysaccharide structure assay was done at the Complex Carbohydrate Research Center at University of Georgia. Samples were partially depolymerized by probe sonication for 30 min at 0°C and de-O-acetylated by adjusting the solution to pH of 11 with concentrated ammonium hydroxide, followed by incubation at 25°C for 20 h and lyophilization.

The samples were deuterium exchanged by lyophilization from D_2_O (99.9% D), dissolved in 0.28 ml D_2_O (99.96% D), and transferred to an NMR tube with susceptibility plugs (Shigemi). A 1D proton NMR spectrum was acquired on a Varian Inova-600 MHz spectrometer at 343 K (70°C). The spectral width was 5,102 Hz, the acquisition time was 1.61 s, and 64 scans were collected. The spectrum was processed using the Mestre-C NMR software. Linear prediction to 16K and a Gaussian function (2 Hz line broadening) were applied to the flame ionization detector (FID) to obtain a full spectrum. Linear prediction to 16K, a negative 3-Hz exponential, and a 1.5-Hz Gaussian function were applied to obtain a resolution-enhanced partial spectrum of the region displaying the mannose anomeric protons. Chemical shifts were measured relative to internal 2,2-dimethyl-2-silapentane-5-sulfonic acid (DSS; *D*_6_ = 0.00 ppm).

The mannose anomeric proton peaks were deconvoluted using the Mestre-C line-fitting routine, and the resulting peak areas were used to estimate the proportions of the mannosyl triads present in the samples.

### ^1^D-NMR analysis of native C. neoformans secreted polysaccharides.

NMR data were collected on a 600-MHz magnet equipped with a Bruker Avance II console and equipped with a triple resonance, TCI cryogenic probe and *z*-axis pulsed-field gradients. All experiments were conducted at 30°C with 64 scans and an FID size of 16,384 points. Standard Bruker pulse sequences were used to collect the 1D data (p3919gp and zggpw5). Data were processed in Topspin (Bruker version 3.5) by truncating the FID to 8,192 points, using a squared cosine bell window function, and zero filling to 65,536 points. Structural reporter groups were defined as previously described (21). Briefly, deconvolution of ^1^H spectra was done using the built-in line-fitting function of MNova. All fitting parameters were left at their default values, and a Lorentzian-Gaussian function was applied to the SRG and acetylation regions of the spectra, 100 iterations were used to get a good convergence of the fit.

### Hydrodynamic radius of polysaccharide samples by dynamic light scattering.

The hydrodynamic radii of capsular polysaccharide and exopolysaccharide were measured by dynamic light scattering in a 90 Plus/BI-MAS multiangle particle-sizing analyzer as previously described (22). The intensity of light scattered due to the random motion of particles was processed by the autocorrelation function *C*(*t*), calculated as *A*^2Γ^ + *B*, to estimate the hydrodynamic diameter of polysaccharide molecules.

### Fluorescence and immunofluorescence microscopy of C. neoformans.

Approximately 10^6^ cells/ml were incubated with a primary capsular monoclonal antibody, IgG 18B7 (51), at 30°C for 1 h with shaking. Cells were washed three times in blocking buffer (1% bovine serum albumin (BSA) in phosphate-buffered saline) and incubated with secondary FITC-labeled anti-mouse IgG and 1 μg/ml Uvitex (cell wall stain) at 30°C with shaking. Cells were washed twice with blocking buffer, pipetted onto a slide, and imaged with an Olympus AX 70 microscope using a 100× objective with oil immersion.

### Enzyme-linked immunosorbent assay.

Antibody reactivities to the EPS and CPS of H99 and the *MAT*α *mio1*Δ*2*Δ*3*Δ strain cultured in glucose, inositol, or both glucose and inositol (1:1) were tested by capture enzyme-linked immunosorbent assay (ELISA). The GXM standard is lyophilized unfractionated (>100-kDa) C. neoformans EPS. This was resuspended with PBS at 1 mg/ml. Ninety-six-well polystyrene microtiter plates were coated with 50 μl of IgM MAb 2D10 at 10 μg/ml and incubated for 1 h at 37°C. Following coating, the plate was blocked with 200 μl of 1% BSA in PBS and incubated for 16 h at 4°C. Fifty microliters of GXM standard and native CPS or EPS fractions was added, and the mixture was serially diluted 1:2 in PBS and incubated 1 h at 37°C. Polysaccharide detection was done with 50 μl IgG1 MAb 18B7 at 2 μg/ml in 1% BSA–PBS and incubated 1 h at 37°C. Detection of bound MAb 18B7 was done with 50 μl of a 1-μg/ml alkaline phosphatase-conjugated goat anti-mouse antibody specific for IgG1 isotype (Southern Biotechnology) suspended in 1% BSA–PBS following 1 h of incubation at 37°C. Plates were washed 3 times after both antibody incubation steps with TBS buffer (10 mM Tris-HCl, 150 mM NaCl, 1 mM NaN_3_, 0.1% Tween 20 [pH 7.4]). Binding reactions were detected by absorbance at 405 nm following the addition of *p*-nitrophenyl phosphate disodium hexahydrate using a microplate reader (Multiskan MS; Labsystems, Helsinki, Finland). By fitting the MAb-PS binding curve as a function of Ab concentration to a one-site total binding model, the binding maxima and dissociation constant were calculated. For all binding curves, the goodness of fit (*R*) was >0.98.

### Murine and rabbit infections.

Virulence of the C. neoformans strains was assessed using both murine infection models and a rabbit meningitis model of cryptococcosis as previously described (12, 52, 53). For virulence study in murine models, female A/Jcr mice (The Jackson Laboratory, ME) with an average weight of 20 to 25 g were used throughout these studies. Animal studies were performed at the Rutgers animal facility. Cryptococcus strains were grown at 30°C overnight; cultures were washed twice with 1× PBS by centrifugation, and cell pellets were resuspended in fresh YNB–1% glucose or YNB–1% inositol liquid culture for 5 days at 30°C with shaking. Groups of 10 mice were used for each infection. For the intranasal inhalation model, mice were intranasally infected with 10^5^ yeast cells of each strain in 50 μl PBS as previously described (54). For the intravenous injection model, 5 × 10^4^ yeast cells in 100 μl volume for each strain were inoculated via tail vein injection. For intracerebral injection model, mice were sedated with a xylazine/ketamine combination, and the top of the head was sterilized using antiseptic until the fur was thoroughly wet. Five hundred yeast cells in 50 μl PBS were injected directly into the cerebrum as previously described (12). Animals that appeared moribund or in pain were sacrificed by CO_2_ inhalation. Survival data from the murine experiments were statistically analyzed between paired groups using the long-rank test and the PRISM program 5.0 (GraphPad Software) (*P* values of <0.01 were considered significant).

In the rabbit model, 2.3- to 2.7-kg male New Zealand White rabbits were immunosuppressed starting 1 day prior to inoculation of yeasts and then daily for the remainder of the study period, with 5 mg/kg hydrocortisone acetate intramuscularly. Rabbits were sedated with ketamine (30 mg/kg) and xylazine (3 mg/kg) and then inoculated with 1 × 10^8^ CFU in 0.3 ml of PBS via direct injection into the cisterna magna. CSF was collected under sedation on days 4, 7, and 9 or 10 postinoculation to perform quantitative CFU counts on YPD-chloramphenicol plates. Rabbit infection experiments were performed at Duke University, in a Division of Laboratory Animal Resources facility. A total of 15 rabbits were tested (9 infected with the *mio1Δ2Δ3Δ* strain and 6 infected with H99) in two separate experiments. Association between treatment group and log_10_ CFU per ml was assessed using a linear mixed-effects model that included terms for treatment group and experiment day and an interaction term for experiment day and treatment group (R 4.1.0 packages lmerTest, version 3.1.3, and emmeans, version 1.6.3).

### Histopathology and fungal burden in infected organs.

Infected mice were sacrificed at the endpoint of the experiment according to the Rutgers University IACUC-approved animal protocol. The lungs and brains were dissected and fixed in 10% formalin solution for section preparation at Rutgers University Histology Core Facility. Tissue slides were treated either with hematoxylin and eosin (H&E) staining for bronchus-associated lymphoid tissue or with Grocott’s methenamine silver (GMS) staining for fungal morphology observation *in vivo*. Infected lungs, brains, and spleens were also isolated and homogenized (Ultra-Turrax T8; IKA) in 3 ml cold 1× PBS buffer for 1 min for each type of organ. The tissue suspensions were serially diluted and plated onto YPD agar medium with ampicillin and chloramphenicol, and colonies were counted after 3 days of incubation at 30°C.
