# Supplementary figures and images for: Inositol Metabolism Regulates Capsule Structure and Virulence in the Human Pathogen Cryptococcus neoformans
Source: mBio. 2021 Nov 2;12(6):e02790-21. doi: 10.1128/mBio.02790-21 (PMC8561382; doi:10.1128/mBio.02790-21)

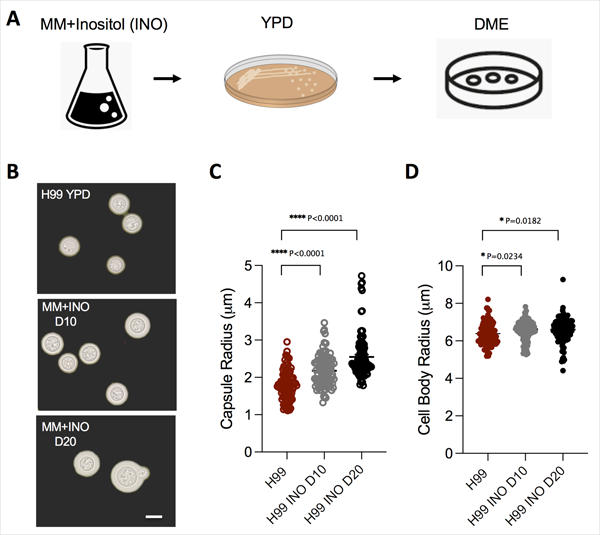

Supplement: FIG S1 [file mbio.02790-21-sf001.tif]

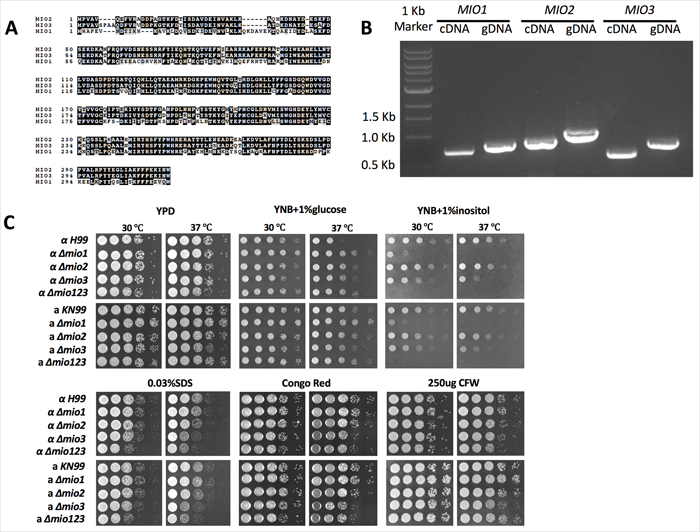

Supplement: FIG S2 [file mbio.02790-21-sf002.tif]

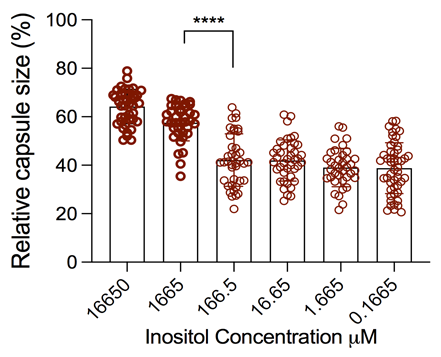

Supplement: FIG S3 [file mbio.02790-21-sf003.tif]

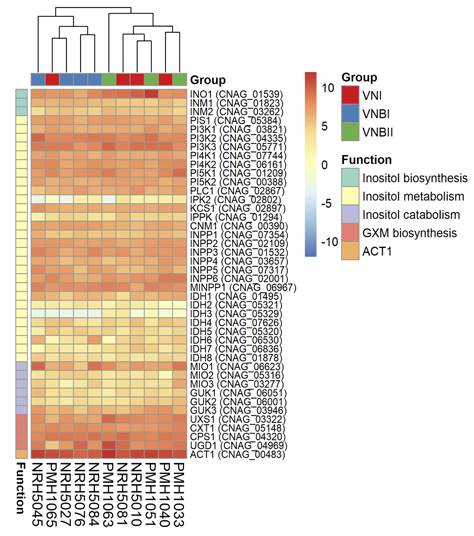

Supplement: FIG S4 [file mbio.02790-21-sf004.tif]
