# Supplementary material for: Inositol Metabolism Regulates Capsule Structure and Virulence in the Human Pathogen Cryptococcus neoformans
Source: mBio. 2021 Nov 2;12(6):e02790-21. doi: 10.1128/mBio.02790-21 (PMC8561382; doi:10.1128/mBio.02790-21)
Supplement: TABLE S1 [file mbio.02790-21-st001.docx]

Supplemental Table S1 Primers used in this study

| Primer name | Sequence (5’-3’) | Description |
| --- | --- | --- |
|  |  |  |
| CX5 | GTAAAACGACGGCCAG | M13F |
| CX6 | CAGGAAACAGCTATGAC | M13R |
| JH8994 | TGTGGATGCTGGCGGAGGATA | JH8994 |
| CX264 | GTTTGTCGCCTTCTTCAGCAT | MIO1 F1 |
| CX271 | CTGGCCGTCGTTTTACGGAATGGCGAATGGACCACAAG | MIO1 R1 |
| CX272 | GTCATAGCTGTTTCCTGTCGCGAAAGAAGACACATACAG | MIO1 F2 |
| CX267 | CGATCTGTAAGAGCGTCTTG | MIO1 R2 |
| CX268 | GACGCTGTCGAGAAGACTCAAG | MIO1 F3 |
| CX269 | CCTTGACAGCCTTGAGCTGTG | MIO1 R3 |
| CX270 | GAACCACCGCTTGGTAGGAAG | MIO1 F4 |
| CX273 | TTGGCTGCTCACCTCTCATTGC | MIO2 F1 |
| CX274 | CTGGCCGTCGTTTTACAGAGTAGAGCCAGAGTGGAGTTG | MIO2 R1 |
| CX275 | GTCATAGCTGTTTCCTGGTCACAGCAGGATCGAGAACAG | MIO2 F2 |
| CX276 | ATGCAACGCCCAATGAGTCGTG | MIO2 R2 |
| CX262 | CGTCGCCAAGCTCAAGGCTCAAC | MIO2 F3 |
| CX263 | GCATCTCACTGCTACAATCACCTC | MIO2 R3 |
| CX277 | GGTTGGAGGTTGACATGCTG | MIO2 F4 |
| CX236 | GTCATGGTGGCTGGGAAAGGT | MIO3 F1 |
| CX237 | CTGGCCGTCGTTTTACTGCAGATGACTGGCGAGAGA | MIO3 R1 |
| CX238 | GTCATAGCTGTTTCCTGGTACTTGTTTGGTGCCAGGCT | MIO3 F2 |
| CX239 | TTCGAGAGCAGGCACAGAAG | MIO3 R2 |
| CX240 | GGTTGCTCATAGTTGGTTGGTAG | MIO3 F3 |
| CX241 | GAAGGTCAGTACAGTTCTACCAC | MIO3 R3 |
| CX553 | GTCGCCCAACCCACAACAACT | MIO3 F4 |
| CX672 | ATCCACTAGTTCTAGAATGCACAGCGAGTCGGAGGAGTAT | MIO1 Comp F |
| CX673 | TGGCGGCCGCTCTAGAAAGTAGTGGCGTCAGCGTTGGTCT | MIO1 Comp R |
| CX49 | TGAGAAGGACCCTGCCAACA | GAPDH F |
| CX50 | ACTCCGGCTTGTAGGCATCAA | GAPDH R |
| CX534 | AAGGACGCTGTCGAGAAGACTCAA | MIO1 QPCR F |
| CX535 | ATTGCTTCCCAGATGGACATGCGA | MIO1 QPCR R |
| CX536 | TTGCTGTTCAGGAATTCGTTCGCG | MIO2 QPCR F |
| CX537 | GGTTTGCTTGGTGTGTTGCTCGAT | MIO2 QPCR R |
| CX538 | TCGCTGTCTCTCCCGCCGCTCAGG | MIO3 QPCR F |
| CX539 | TGTTCGATGTAAAAGCGTCGCGAA | MIO3 QPCR R |
| CX1891 | CCGATTCGGTTTCGACTCTAC | UXS1 QPCR F |
| CX1892 | GCACCACCAGTAACCAAGAT | UXS1 QPCR R |
| CX1935 | CCATCCACGATCAACCATCTAT | CXT1 QPCR F |
| CX1936 | GGGTGTCAGAGTTCTTCTCTTC | CXT1 QPCR R |
| CX668 | AGGCTCGTATCGCTGTCTA | UGD1 QPCR F |
| CX669 | CGTAGATCTTCTTCCAGTCAAGAG | UGD1 QPCR R |
| CX532 | ACACACATTGACGGTGGTATCCCT | CPS1 QPCR F |
| CX533 | TTTGCAGACCTGGACGTAGGTGTT | CPS1 QPCR R |
| CX700 | CAGAAGTGGACGACCTTCAATC | GUK1 QPCR F |
| CX701 | CCTGATCCAGGAACGCTTATAAAT | GUK1 QPCR R |
|  |  |  |
